# Non-Destructive Discrimination of Sunflower Seeds with Different Internal Mildew Grades by Fusion of Near-Infrared Diffuse Reflectance and Transmittance Spectra Combined with 1D-CNN

**DOI:** 10.3390/foods12020295

**Published:** 2023-01-08

**Authors:** Jie Liu, Shuang Fan, Weimin Cheng, Yang Yang, Xiaohong Li, Qi Wang, Binmei Liu, Zhuopin Xu, Yuejin Wu

**Affiliations:** 1 Hefei Institutes of Physical Science, Chinese Academy of Sciences, Hefei 230031, China; 2 Science Island Branch, Graduate School of USTC, Hefei 230026, China

**Keywords:** near-infrared spectroscopy, fusion spectra, 1D-CNN, sunflower seed, internal mildew

## Abstract

Internally mildewed sunflower seeds, which cannot be recognized and discarded based on their appearance, pose a serious risk to human health. Thus, there is a need for a rapid non-destructive mildew grade discrimination method. Currently, few reports are available regarding this process. In this study, a method based on the combination of the near-infrared diffuse reflectance and near-infrared diffuse transmission (NIRr-NIRt) fusion spectra and a one-dimension convolutional neural network (1D-CNN) is proposed. The NIRr-NIRt fusion spectra can provide more complementary and comprehensive information, and therefore better discrimination accuracy, than a single spectrum. The first derivative (FD) preprocessing method could further improve the discrimination effect. By comparison against three conventional machine learning algorithms (artificial neural network (ANN), support vector machine (SVM), and K-nearest neighbor (KNN)), the 1D-CNN model based on the fusion spectra was found to perform the best. The mean prediction accuracy was 2.01%, 5.97%, and 10.55% higher than that of the ANN, SVM, and KNN models, respectively. These results indicate that the CNN model was able to precisely classify the mildew grades with a prediction accuracy of 97.60% and 94.04% for the training and test set, respectively. Thus, this study provides a non-destructive and rapid method for classifying the mildew grade of sunflower seeds with the potential to be applied in the quality control of sunflower seeds.

## 1. Introduction

Sunflower seeds, one of the four largest oil crops and a major source of vegetable oil worldwide, contain many nutrients, including unsaturated fatty acids, proteins, human-essential amino acids, fiber, and vitamins [1]. To prevent microbial contamination, sunflower seeds are typically stored, transported, and marketed as whole seeds with their shells intact. In China, shelled sunflower seeds are usually eaten directly or used for the extraction of edible oil [2]. However, shelled sunflower seeds are prone to develop internal mildew, meaning that the kernels become moldy with observable mildew spots inside the shell. Unfortunately, most internally moldy sunflower seeds, without external evidence, cannot be recognized using manual sorting or color sorting equipment. For this reason, accidental ingestion is frequent, negatively affecting human health and consumer experience [3,4].

As mold contamination is a gradual process, timely intervention could reduce economic loss; however, sunflower seeds with different mildew degrees do have their own uses, e.g., slightly moldy seeds can be detoxified and fed to animals [5]. Thus, the grading and sorting of sunflower seeds is of great significance for guaranteeing product quality and economic value. Manual sorting, the traditional detection method, has several disadvantages, including a low efficiency and a high rate of missed detection. Color sorting technology is an effective technology to identify and sort external mildew or defected seeds [6]. Unfortunately, most internally moldy shelled sunflower seeds, which have a normal-looking shell and do not show external evidence of mildew, cannot be recognized and discarded using color sorting equipment. Thus, there is a need to develop a rapid, precise, and non-destructive method for detecting internally moldy whole sunflower seeds.

Recently, various types of imaging and spectral sensor technologies [7,8] have been applied to the non-destructive sorting of grains. Among them, near-infrared spectroscopy (NIRS) has been widely used due to its strong penetrability, high efficiency, and simultaneous analysis of multiple components [9]. For nuts with thicker and harder husks, only a few reports are available regarding the identification of internal mildew [10,11], with the available studies mainly focusing on internal hollowness, defects, insect pests, and nutrients based on NIRS technology [12,13,14]. Additionally, very few reports are currently available regarding the discrimination of internal mildew grades inside nuts, especially small nuts (e.g., sunflower seeds), because differences among the spectra of different mildew grades are weak, with near-infrared light intensity weakened when passing through the shell. Thus, it is imperative to improve the discrimination accuracy.

Near-infrared diffuse reflectance (NIRr) and near-infrared diffuse transmission (NIRt) are two common NIR spectroscopic methodologies. Since the two types of spectra have their own advantages [15,16], the NIRr-NIRt data fusion method is a promising alternative to achieve high discrimination accuracy based on the complementary or enhanced signals of the two spectra. Our previous study on the determination of rice flour constituents showed that the data fusion method has higher detection accuracy than NIRr or NIRt alone [17]. A one-dimension convolutional neural network (1D-CNN) can be used to analyze the NIRr-NIRt fusion spectral data. This algorithm, originally derived from 2D image data analysis [18], can be applied to spectral analysis [19,20] with strong feature extraction and learning abilities, weight sharing, and no need for the manual selection of features. Thus, the NIRr-NIRt data fusion method combined with a 1D-CNN represents a promising technique for the classification of the internal mildew grades of whole sunflower seeds.

The following summarizes the main objectives of this work: (1) to explore the optimal pretreatment conditions for the fusion of NIRr and NIRt data; (2) to construct a 1D-CNN model based on NIRr-NIRt fusion spectra and to verify its superiority over the single spectra; and (3) to verify the superiority of the 1D-CNN model based on the NIRr-NIRt data fusion method over other traditional methods.

## 2. Materials and Methods

### 2.1. Materials

The samples were obtained from a batch of shelled sunflower seeds with high water content from Qiaqia sunflower seed factory in Bayan Nur, Inner Mongolia, in 2020. The collected samples were immediately placed in presterilized polyethylene bags and stored in freezers at −20 °C until further use.

**Figure 1:**
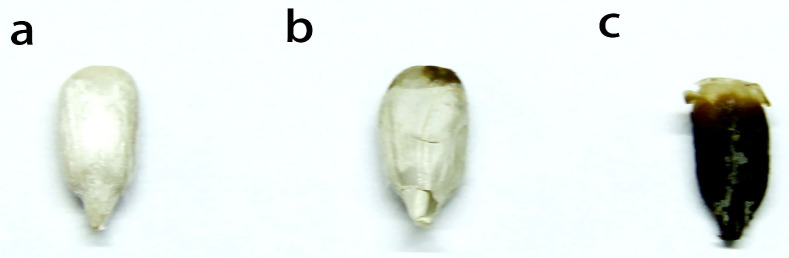
Sunflower seed kernels at different mildew grades: (**a**) normal, (**b**) slightly moldy, and (**c**) seriously moldy.

Sunflower seeds with shells were used to collect spectra. Then, they were hulled and manually assessed at three mildew grades (normal, slightly moldy, and seriously moldy) by several professional experts using national standards [21]. The characteristics of the different mildew grades are shown in Table 1 and Figure 1. After this evaluation, 252 normal samples, 154 slightly moldy samples, and 254 seriously moldy samples were obtained for spectral analysis. During spectral analysis, the Kennard–Stone method [22] was used to separate the datasets of each mildew grade into a training set and a test set at a ratio of 2:1.

### 2.2. Verification of the Mildew Grade

Mid-infrared (MIR) spectroscopy, which characterizes molecular functional groups, is used by the US Food and Drug Administration to determine chemical composition, while the plate colony counting method counts the microorganisms on the surface of the crops [23]. In seeds, mildew infection is a gradual process, and the mildew phenotype is closely related to the change in the biomass and metabolites. Thus, MIR spectroscopy and the plate colony counting method were used to verify the rationality of the visual grading standard. Each type of collected sunflower seed kernel sample (normal, slightly moldy, and seriously moldy) was reduced to about 3 g using the quarter method to guarantee a representative sample [24].

When using MIR spectroscopy to analyze the chemical composition, about 1 g of sunflower seed kernels with different mildew grades were fully crushed and mixed well in liquid nitrogen, followed by freeze drying (FD-1A-50 freeze dryer; Shanghai Bilang Instrument Co., Ltd. Shanghai). The samples for MIR measurement were prepared by mixing 2 mg of freeze-dried sunflower seed kernel powder with 150 mg of dried potassium bromide, followed by pressing under a pressure of 15 MPa for 3 min to obtain a disk pellet. The samples were then subjected to MIR measurement (Bruker Optics GmbH, Ettlingen, Germany) with the spectral range of 4000–400 cm^−1^, a resolution of 4 cm^−1^, and with 64 scans per sample. The results were then analyzed using OPUS 7.0 data processing software. Before the spectral data analysis, all the spectra were pretreated by vector normalization and baseline correction. Consistency with the visual results was determined by comparing the differences between MIR spectra and characteristic peaks at different mildew grades. 

When performing the microbial counts, 2 g of kernels at each mildew grade were mixed with 200 mL of sterile water in a shaker bottle and incubated for 20 min with shaking at 200 rpm. Each dilution was plated on Rose Bengal medium supplemented with 30 g/L sodium chloride; this separation medium can reduce the growth of filamentous fungi. The plates were observed after culture for 7 days in the dark at 28 °C. Consistency with the visual results was determined by comparing the differences in the colony number at different mildew grades.

### 2.3. NIR Spectra Collection

Before spectra collection, sunflower seeds were placed in the dryer to balance the moisture. In this study, all spectra were collected using a MPA Fourier transform near-infrared spectrometer (Bruker, Ettlingen, Germany), which can be operated under two measurement modes: NIRr and NIRt. The measurement conditions are as follows: one single shelled sunflower seed was placed in the sample window above the light source. Then, the NIRr spectrum was measured with the diffuse reflection mode, and the NIRt spectrum was measured with the diffuse transmission mode. In each mode, the spectrum was collected once on the front and back side of the shelled seed. The final spectrum of each sample was obtained by averaging both sides. To ensure data quality, the spectral variables greater than 10,000 cm^−1^ in NIRr and NIRt (e.g., greatly interfered by noise) were removed. The ranges of the spectra recorded under NIRr and NIRt were 3996–10,000 cm^−1^ (1000–2502 nm) and 5793–10,000 cm^−1^ (1000–1726 nm), respectively, with a resolution of 16 cm^−1^. Each acquired spectrum was the average of 64 scans and represented in absorbance (A = logR^−1^). Lastly, the NIR data were analyzed using OPUS software (Bruker Optik GmbH, Ettlingen, Germany). 

### 2.4. Evaluation of Spectral Fusion of NIRr and NIRt and Optimization of Fusion Conditions

To eliminate any interference caused by spectral baseline drift or the scattering effects by particle size difference, as well as the inconsistency of the absorbance and morphology between NIRr and NIRt spectra during fusion, first derivative (FD) and standard normal variable transformation (SNV) were used for the fusion spectra of NIRr and NIRt and the single spectra. For FD, the Savitsky–Golay method was used with the number of smoothing points as 17 and the polynomial order as 2, and no preprocessing (NP). The appropriate preprocessing method was selected by comparative analysis under the 1D-CNN model. Additionally, zero mean normalization (Z-score) was taken before preprocessing for all the spectra.

### 2.5. 1D-CNN Discriminant Model Construction

The basic architecture of the 1D-CNN model was mainly structured as an input layer, two convolutional layers, two pooling layers, one flattening layer, two fully connected layers, and an output layer (softmax classifier). In this study, the fusion and single spectral data were used as the input and the predicted classification result was used as the output. This model employed two convolutional layers, each followed immediately with a pooling layer, which can reduce the output size and risk of overfitting. The flattening layer was used to flatten the multidimensional input data into 1D data as the transition from the convolutional layer to the fully connected layer. The fully connected layer was then applied to provide 1D data for the softmax classifier. By connecting the softmax classifier, the classification probability of the near-infrared data was calculated. 

In our research, in order to establish the reliable model consistent with the real situation, we adopted the samples with naturally occurring mildew because, in China, the internal mildew of sunflower seeds mainly occurs naturally in the field [25]. Although more artificial mildew samples can be obtained via artificial humidification, data from these samples could not fully correspond to the real state. However, it is difficult to obtain a large number of samples due to the low occurrence rate of internal mildew; thus, the potential risk of overfitting was taken into consideration. To address this, in addition to the design of the architecture of the 1D-CNN model, the ReLU function and dropout method were adopted, as they can effectively reduce overfitting by enhancing generalization ability. Furthermore, several key parameters were also adjusted to obtain the optimized model for all the fusion and single spectral datasets based on the reliability and the discrimination accuracy. The main parameter settings of the 1D-CNN model are shown in Table 2. For all the datasets, the 1D-CNN models were randomly trained and tested eight times, and the average and standard deviation of these tests were used as the final result. The accuracy and loss function were used to diagnose the models.

### 2.6. Conventional Classification Methods

For comparison with the 1D-CNN model, three commonly used machine learning algorithms, namely, artificial neural network (ANN) [26], support vector machine (SVM) [27], and K-nearest neighbor (KNN) [28], were used to classify the fusion spectra. To obtain reliable results, the three classification algorithms were randomly trained and tested eight times, and the mean prediction accuracy (PA) and standard deviation (SD) of these tests were used as the final result. The parameters used in the model are summarized in the following sections.

#### 2.6.1. ANN

The fusion spectral data of the training set samples were imported into the artificial neural network of MATLAB, with each layer adopting the sigmoid transfer function, a target error of 0.001, a learning rate of 0.1, and 1000 as the number of training iterations. Here, the values 0, 1, and 2 represent the normal, slightly moldy, and seriously moldy samples, respectively. The deviation threshold was set to 0.5, and the recognition result was determined to be accurate when the difference between the true value and the predicted value was within the range of 0.5. The network was also optimized by adjusting the number of neuron nodes in the hidden layer based on the discrimination accuracy. After optimization, the number of neuron nodes was set to 100.

#### 2.6.2. SVM

The radial basis function (RBF) was used as the kernel function, and the sigmoid function was selected as the excitation function. The penalty parameter (c) was also adjusted to achieve the highest classification recognition rate. After optimization, the penalty factor was set to 0.8.

#### 2.6.3. KNN

The model was also optimized by adjusting the number of neighbors based on the discrimination accuracy. After optimization, the number of neuron neighbors was set to 3.

### 2.7. Model Evaluation Method and Software

The performance of the classification model was comprehensively evaluated by the joint use of the mean prediction accuracy (PA), mean F1 score, and SD. A higher PA and F1 score and a smaller SD were associated with a better performance of the model. Among these, the F1 score was the mean value of the weighted F1 score of three categories. PA was calculated as follows (Equation (1)):

PA = (Nc/N) × 100%(1)

where N denotes the total number of samples and Nc denotes the number of samples predicted to be real.

All data preprocessing and ANN calculations were performed using MATLAB 2015b (MathWorks, Inc., Natick, MA, USA). The training and testing of 1D-CNN, SVM, and KNN were all implemented in Python (3.8.8) using the Keras library (v2.4.3) and TensorFlow (v2.3.0).

## 3. Results

### 3.1. Verification of the Mildew Grade

#### 3.1.1. Microbial Detection

**Figure 2:**
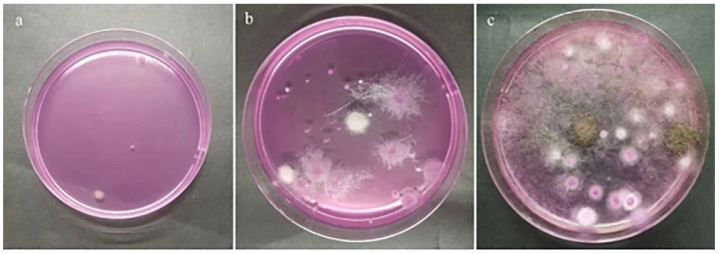
Plates used for microbial counts of different mildew grades of sunflower seed kernels: (**a**) normal, (**b**) slightly moldy, and (**c**) seriously moldy.

The plate colony counting method was used to identify mold-infected sunflower seeds with different mildew grades. The analysis results, as shown in Figure 2, indicate that the microbial counts were consistent with the results from visual inspection.

#### 3.1.2. MIR Detection

**Figure 3:**
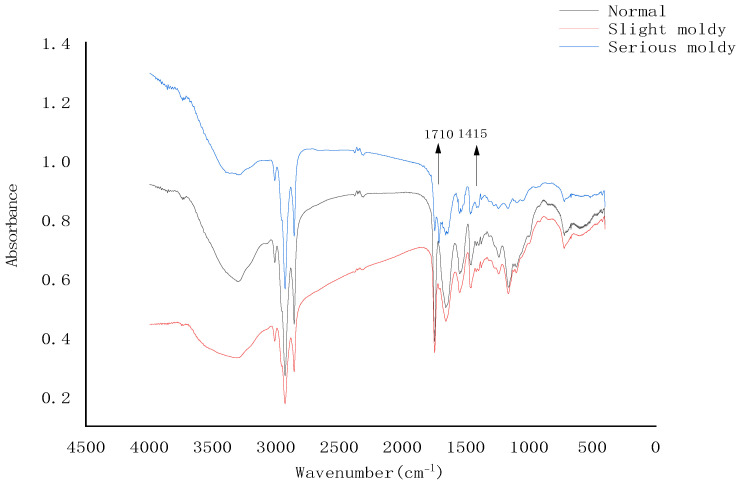
Mid-infrared spectra of sunflower seed kernels with different mildew grades.

Samples with different mildew grades were evaluated by comparing their MIR spectra, as shown in Figure 3. Prominent differences in intensity were observed for the peaks at about 1710 cm^−1^ and 1415 cm^−1^, which were closely related to the mildew grade. Seriously moldy and slightly moldy samples both had a sharp peak at 1710 cm^−1^, and the peak intensity of seriously moldy samples was significantly higher than that of the slightly moldy samples, while normal samples had no peak at 1710 cm^−1^. The normal and slightly moldy samples both had a peak at 1415 cm^−1^, while the seriously moldy samples had no such peak. Bands at 1710 cm^−1^ and 1415 cm^−1^ corresponded to the C=O bending and C-N bending of fat and protein, respectively, for sunflower seed kernels contain about 50% fat and 30% protein. We conclude that fat produces a large number of small molecular ketones and aldehydes under the action of mold lipase and lipoxygenase, while protein produces peptides and amino acids under the action of mold protease. As a result, C=O bending appeared and C-N bending disappeared after mildew infection.

Above all, the results show that the microbial count and MIR spectra of the samples with different mildew grades were markedly different, consistent with the results from visual inspection, indicating the reliability of the visual inspection standard. It was precisely because of the differences in the microbial count and metabolites within the kernels that NIR could capture enough information to build a discriminant model.

### 3.2. Fusion Spectra Analysis under Different Pretreatment Conditions

**Figure 4:**
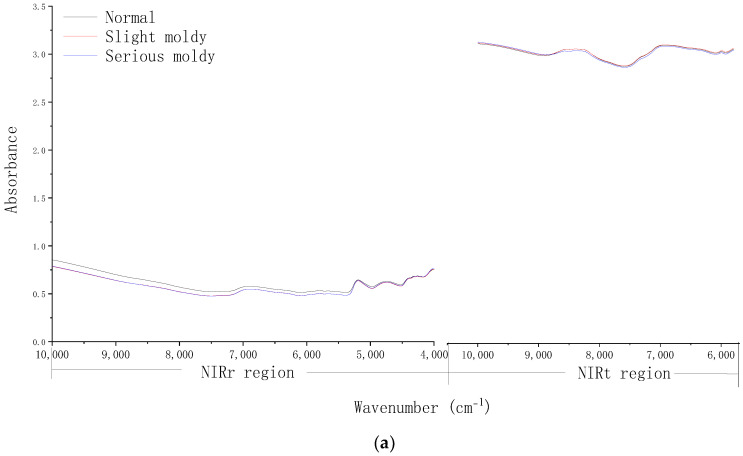

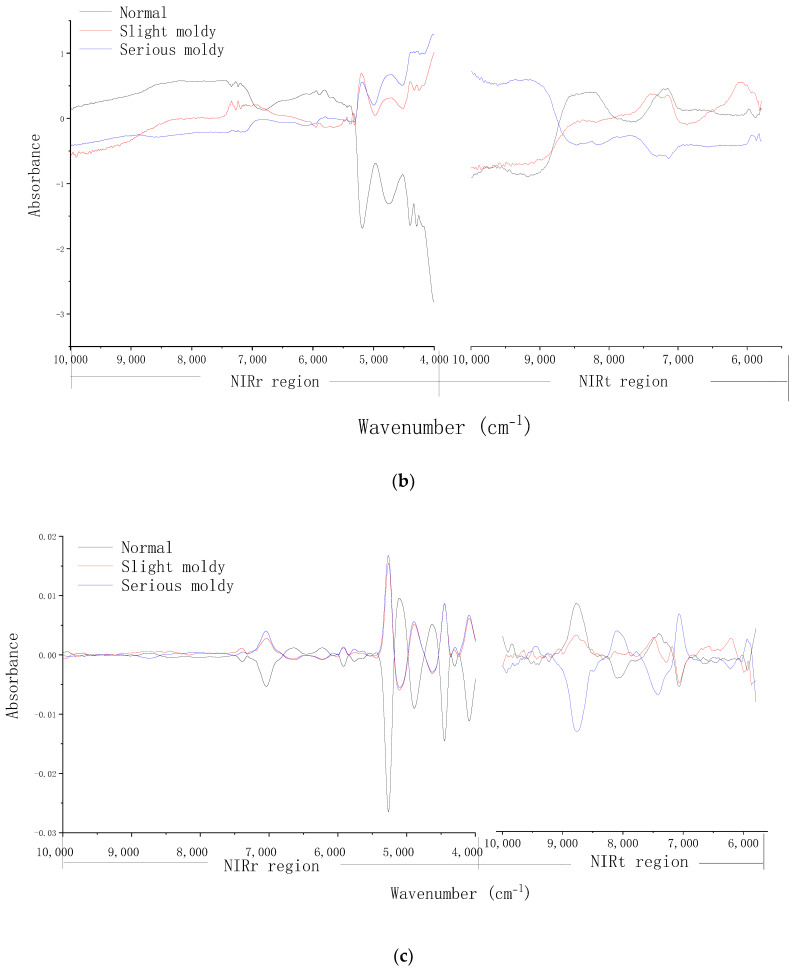
Fusion spectra of NIRr and NIRt under different pretreatment conditions: (**a**) NP, (**b**) SNV, and (**c**) FD. Abbreviations: NIRr, near-infrared diffuse reflectance; NIRt, near-infrared diffuse transmission; FD, first derivative; SNV, standard normal variable transformation; NP, no preprocessing.

The mean fusion spectra of NIRr and NIRt under different pretreatment conditions are shown in Figure 4. The raw spectra of different mildew grades closely overlapped, and thus do not show differences among different grades of mildewed samples. The NIRr and NIRt spectral fingerprint exhibited different absorbance values. At 10,000–7500 cm^−1^ (1000–1333 nm), an absorption peak corresponding to C-H stretching (second overtone) in the region of 8264–8696 cm^−1^ (1150–1210 nm) can be observed in the NIRt region, but only a relatively flat curve in the NIRr region. The range of the NIRr spectrum at 3996–5793 cm^−1^ (1726–2502 nm) consists of a large number of absorption peaks closely related to protein and fat, e.g., C-H stretching (first overtone) at 5555–5882 cm^−1^ (1700–1800 nm), C-H stretching (combination tone) at 4347–4166 cm^−1^ (2300–2400 nm), N-H stretching (combination tone) at 4878–4854 cm^−1^ (2050–2060 nm), and C=O bending (second overtone) at around 5263 cm^−1^ (1900 nm). However, this range is not included in the NIRt spectrum. Additionally, the absorbances recorded in the NIRt spectra were higher than those recorded in the NIRr spectra due to the thickness of the kernel, leading to less NIRt light returned to the sensor. Due to the differences in the absorbance, there was a noticeable gap at the splicing site of the NIRr and NIRt spectra. 

After SNV treatment (Figure 4b), the gap between the NIRr and NIRt spectra was reduced, and the difference between the different mildew grades was enhanced to some extent, which could be found between regions with characteristic absorption. In NIRr exclusive region of 3996–5793 cm^−1^ (1762–2502 nm), several absorption peaks, including a peak corresponding to C=O bending (second overtone) around the region of 5263 cm^−1^ (1900 nm) and N-H stretching (combination tone) 4878–4854 cm^−1^ (2050–2060 nm) were observed. At 10,000–7500 cm^−1^ (1000–1333 nm), several absorption peaks of the NIRt region were observed, which also corresponded to fat and protein, e.g., an absorption peak corresponding to C-H stretching (second overtone) in the region of 8264–8696 cm^−1^ (1150–1210 nm), but in the NIRr region, the curve was relatively flat with no obvious difference between the different mildew grades. 

For the NIRr-NIRt (FD) spectrum (Figure 4c), the gap at the splicing site between the NIRr and NIRt spectra could be effectively reduced, with the absorbance oscillating around zero. Additionally, a significant difference among the different mildew grades was found between regions with strong characteristic absorption, which showed obvious peaks and valleys, while the absorbance values in other regions were approximately zero. As shown in Figure 4c, in the range spanning 3996–7000 cm^−1^ (1428–2502 nm) of the NIRr region, especially in its exclusive region 3996–5793 cm^−1^ (1762–2502 nm), several strong absorption peaks, including a peak corresponding to C=O bending (second overtone) in the region of 5263 cm^−1^ (1900 nm), and a peak corresponding to C-N bending (second overtone) in the region of 5208 cm^−1^ (1920 nm), were observed. This region exhibited absorption bands corresponding to fat and protein. Meanwhile, only flat curves in the NIRt region of 5793–7000 cm^−1^ (1428–1762 nm) were observed. Multiple absorption peaks in the region 7000–10,000 cm^−1^ (1000–1428 nm) of the NIRt region were observed, which also corresponded to fat and protein. Furthermore, peaks corresponding to C-H stretching (second overtone) in the region of 8200–8300 cm^−1^ (1204–1219 nm) and N-H stretching (second overtone) in the region of 9850–10,000 cm^−1^ (1000–1015 nm) were observed, while the spectral curve of NIRr in the region of 7000–10,000 cm^−1^ (1000–1428 nm) was relatively flat [29]. This shows that the spectral quality of NIRr is better than NIRt over the long wavelength range, and vice versa during the short wavelength range, which can be observed in Figure 4a,b.

Therefore, it can be concluded that FD strengthens the difference among the spectra from sunflower seeds with different mildew grades, which was weak due to the blocking of the shell and the small size of the sunflower seeds, in order to establish a discrimination model.

### 3.3. 1D-CNN Discrimination Model under Different Optimization Conditions

As shown in Table 3, under different pretreatment conditions, the classification effect of the fusion spectrum of NIRr and NIRt was better than that of the NIRr model and NIRt model under the same conditions. Fusion spectra provided a more comprehensive spectral signal than single spectra. Additionally, the fusion spectral model under FD pretreatment gave rise to the best and most robust classification effect, with a mean PA of 94.04%, a mean F1 score of 93.93%, and the lowest standard deviation. These results were also consistent with the spectra presented in Figure 4. 

**Figure 5:**
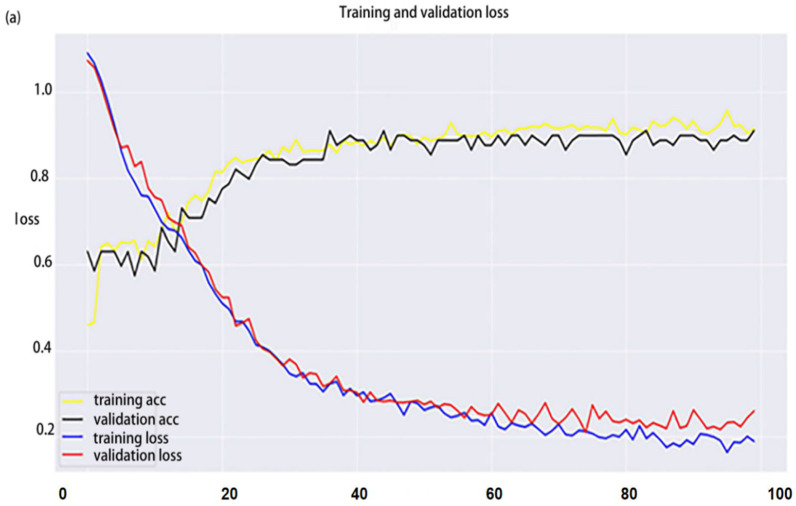

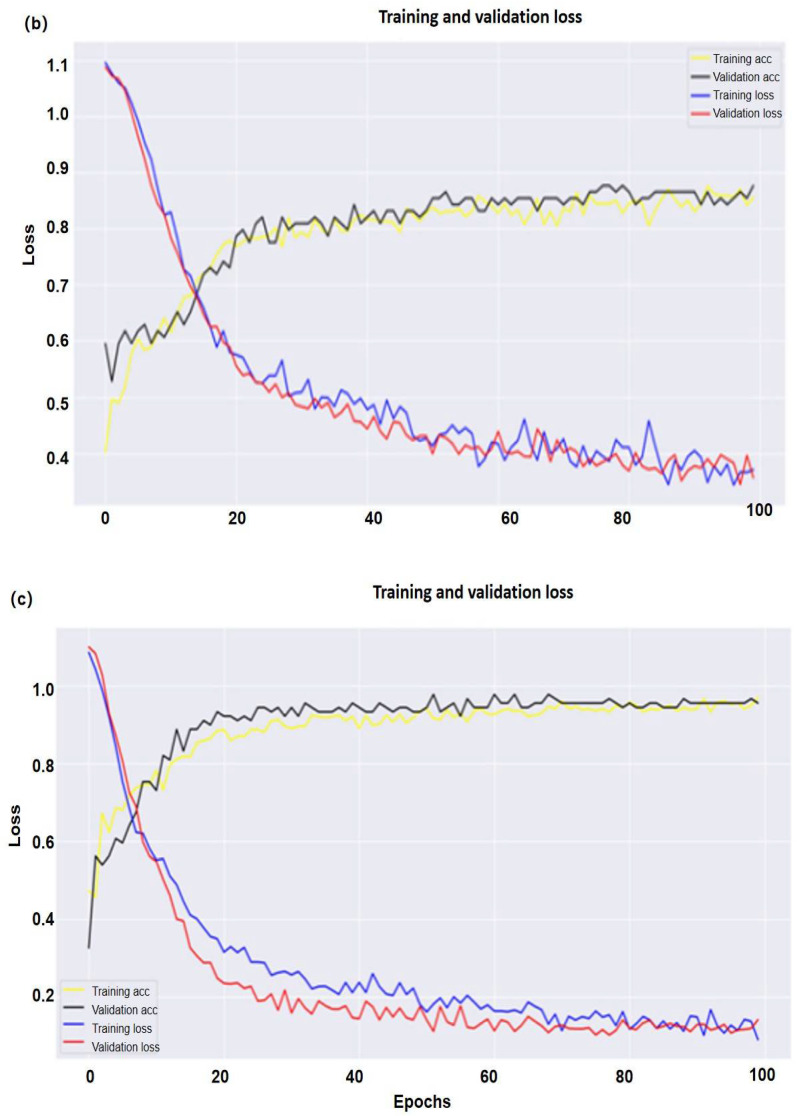
Discrimination accuracy and loss function across epochs of 1D-CNN model: (**a**) NIRr, (**b**) NIRt, and (**c**) NIRr-NIRt. Abbreviations: 1D-CNN, one-dimension convolutional neural network; NIRr, near-infrared diffuse reflectance; NIRt, near-infrared diffuse transmission.

The accuracy and loss function values of the models under FD pretreatment are shown in Figure 5. With an increase in the epoch number, the accuracy of the test and training sets of the three CNN models reached stability with the accuracy of fusion spectra higher than for single spectra, and exhibited a quick convergence of the loss function value. For all the models, the overfitting phenomenon was not significant, and the results are reliable. Additionally, the model also showed good robustness and a strong generalization ability overall.

**Figure 6:**
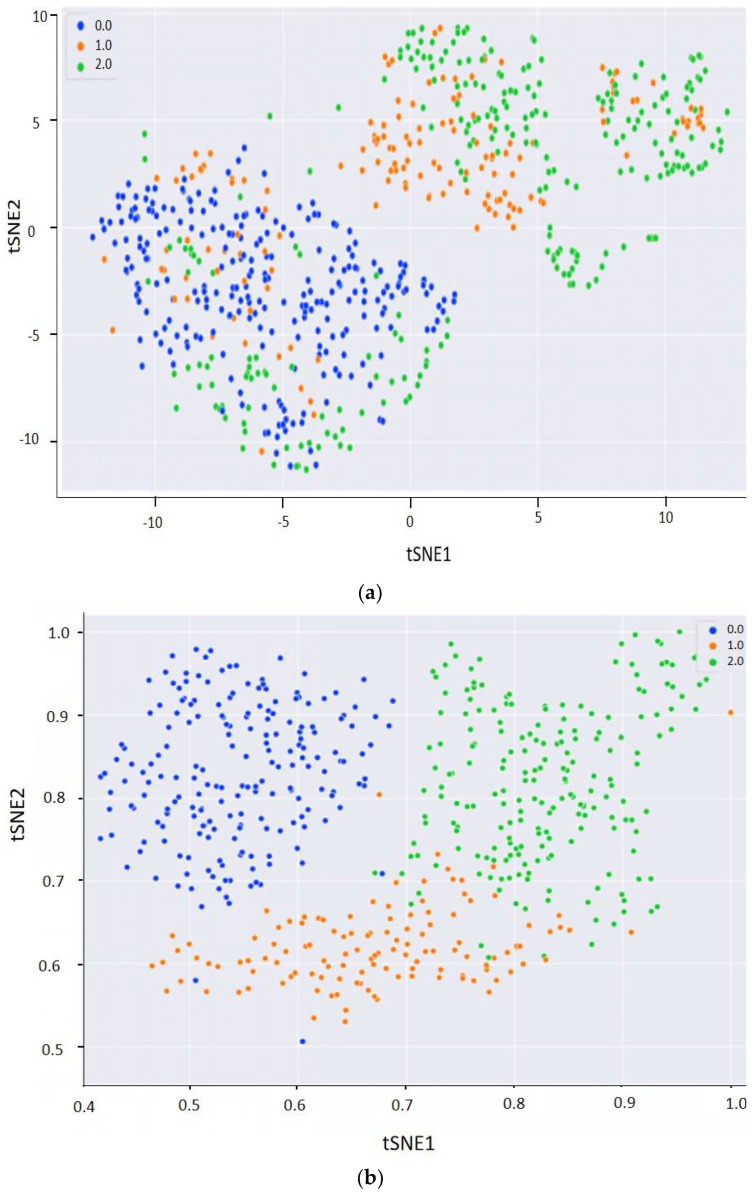

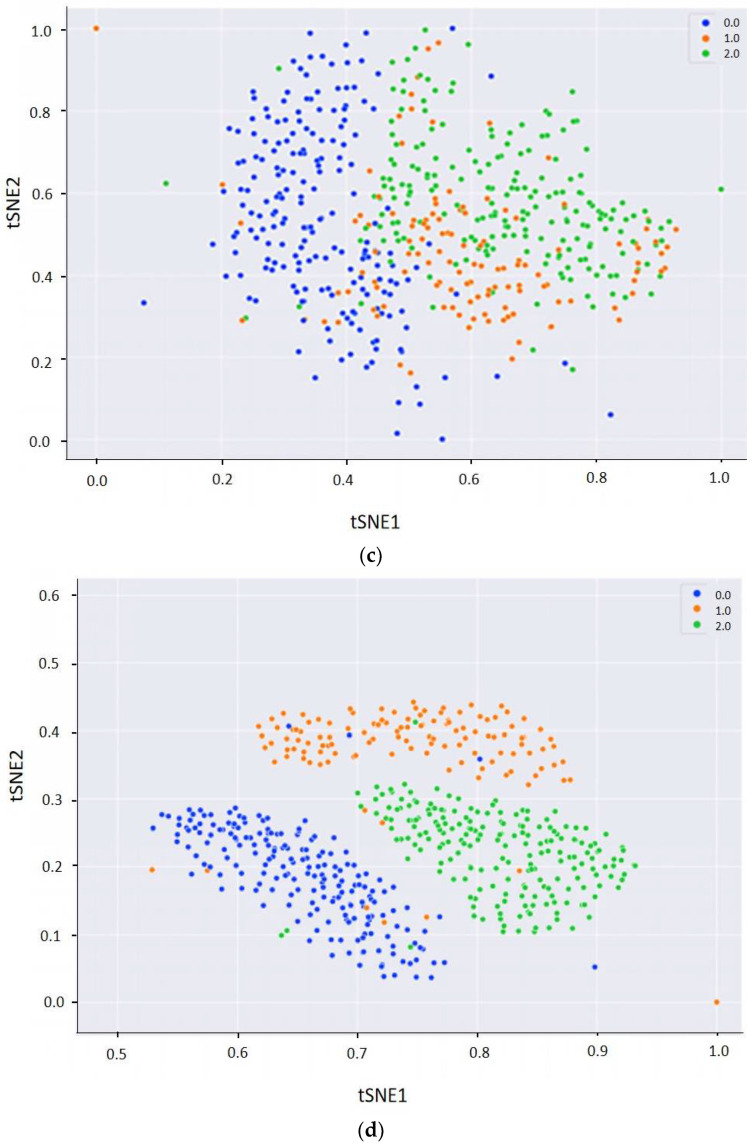
Visualization maps of sunflower seeds using 1D-CNN and t-SNE (0, normal; 1, slightly moldy; and 2, seriously moldy): (**a**) t-SNE before CNN, (**b**) t-SNE after CNN and NIRr, (**c**) t-SNE after CNN and NIRt, and (**d**) t-SNE after CNN and NIRr-NIRt fusion spectra. Note: x and y axes represent two dimensions. Abbreviations: 1D-CNN, one-dimension convolutional neural network; NIRr, near-infrared diffuse reflectance; NIRt, near-infrared diffuse transmission; t-SNE, t-distributed stochastic neighbor embedding.

The t-distributed stochastic neighbor embedding (t-SNE) method is a technique used for nonlinear dimensionality reduction to visualize high-dimensional data in low-dimensional space, while maintaining high-dimensional characteristics [30]. In our study, the t-SNE method was adopted to visualize the features of the layers by giving each data point a location on a two-dimensional map, and to intuitively demonstrate the effectiveness of the classification model. As shown in Figure 6a, for NIRr-NIRt fusion spectra, the t-SNE scatter plots of different mildew grades were mixed and overlapped before adopting 1D-CNN. After adopting the 1D-CNN model combined with NIRr (Figure 6b) or NIRt (Figure 6c), the separation degree increased with confusion among different mildew grades to some extent. Furthermore, the separation degree of NIRr was higher than that of NIRt. Clear boundaries can be observed between the visualization data obtained from the 1D-CNN model of NIRr-NIRt fusion spectra (Figure 6d), indicating that the fusion spectra contained more effective features. This visual result is also consistent with the results presented in Table 3. Therefore, this method has the potential to explain the effectiveness of fusion spectra combined with 1D-CNN for the establishment of a rapid visual classification method.

Thus, under the effective extraction of features by the 1D-CNN, the NIRr-NIRt data fusion method achieved a more satisfactory complementary effect, and therefore, better discrimination accuracy, than single spectra.

### 3.4. Comparison of the 1D-CNN Model with Conventional Classification Algorithms

To evaluate the performance of the CNN model, the ANN, SVM, and KNN models of NIRr-NIRt fusion spectra were established for comparative analysis. The training and testing results of the models are shown in Table 4.

As shown in Table 4, the mean prediction accuracy and the mean F1 value for both training and test sets of the 1D-CNN model were higher than those of other three machine learning models. The mean prediction accuracy of the 1D-CNN model for the training set increased by 1.0%, 5.52%, and 6.2% compared to the ANN, SVM, and KNN models, respectively. Furthermore, the mean prediction accuracy of the 1D-CNN model for the test set increased by 2.01%, 5.97%, and 10.55% compared with the ANN, SVM, and KNN models, respectively. For F1 score, the 1D-CNN model also showed an advantage over the other models. These results indicate that the 1D-CNN model produced the best classification results. 

### 3.5. Discrimination Effect of Sunflower Seeds at Specific Mildew Grade

**Figure 7:**
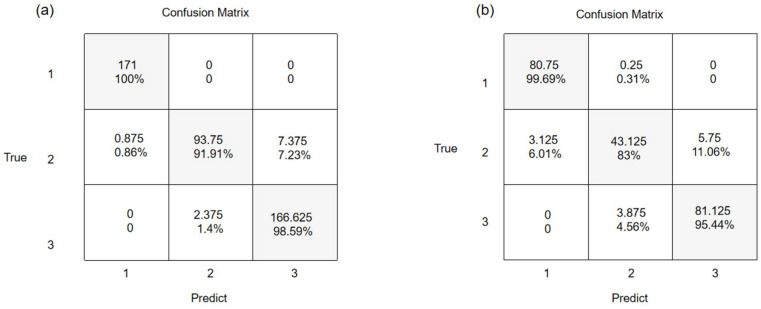
Confusion matrix of NIRr-NIRt fusion spectral 1D-CNN model: (**a**) training set and (**b**) test set (1, normal; 2, slightly moldy; and 3, seriously moldy). The value given in the confusion matrix represents the average of eight times for the training or test sets. Percentages were calculated as the ratio of samples predicted to be true to real samples at specific mildew grade. Abbreviations: 1D-CNN, one-dimension convolutional neural network; NIRr, near-infrared diffuse reflectance; NIRt, near-infrared diffuse transmission.

The mean confusion matrices of the fusion spectral 1D-CNN for the training and test sets are shown in Figure 7. The prediction accuracy of the normal sample was the highest (99.69%), followed by the seriously moldy (95.44%) and slightly moldy samples (83%), the latter of which being the lowest. The results show that the slightly moldy samples were more difficult to discriminate than the samples at other mildew grades. In addition, the proportion of slightly moldy samples misjudged as normal samples (6.01%) was much lower than that of the slightly moldy samples misjudged as seriously moldy samples (11.06%), indicating that slightly moldy and seriously moldy samples are prone to being confused.

**Figure 8:**
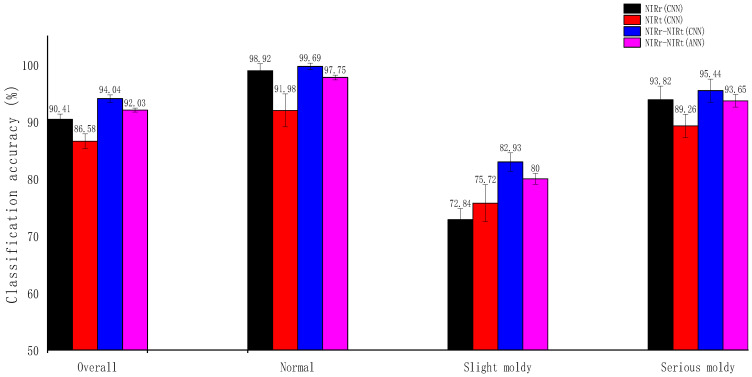
Overall prediction accuracy and prediction accuracy at specific mildew grade using the four different evaluation methods. Abbreviations: CNN, convolutional neural network; ANN, artificial neural network; NIRr, near-infrared diffuse reflectance; NIRt, near-infrared diffuse transmission.

To evaluate the classification accuracy at the specific mildew grade, the fusion spectral 1D-CNN model was compared with the single spectral 1D-CNN and fusion spectral ANN model, which had the highest overall classification accuracy among the machine learning methods evaluated (Figure 8). As shown in Figure 8, for 1D-CNN, the fusion spectra exhibited the best classification performance at all mildew grades. For the discrimination accuracy of normal and seriously moldy samples, NIRr was better than NIRt. However, in terms of the discrimination accuracy of the slightly moldy samples, NIRt was better than NIRr. After spectral fusion, the discrimination accuracy at specific mildew grade all increased, with the slightly moldy samples increasing the most significantly. For the fusion spectral ANN model, the prediction accuracy rate of the normal, slightly moldy, and seriously moldy samples decreased by 1.94%, 2.93%, and 1.79% compared to fusion spectral CNN model, respectively, demonstrating the superiority of CNN over traditional machine learning algorithms.

## 4. Discussion

Previous studies have investigated non-destructive identification methods of internal mildew based on NIRs. Hu et al. [10] used near-infrared diffuse reflectance spectroscopy to identify normal and mildewed chestnuts, and the prediction accuracy was 100% and 96.37%, respectively. Similarly, Zhou [11] established a discrimination model for normal, surface moldy, and internal moldy chestnut based on NIRS with prediction accuracies of 94.74%, 94.44%, and 92.31%, respectively. However, to date, few reports are currently available regarding the discrimination of internal mildew grades inside the sunflower seeds due to the lack of high-precision discrimination method.

The data fusion method is a promising alternative based on the complementary or enhanced signals. Common multisensor data fusion technologies [31,32,33] have to be realized based on different instruments. However, the NIRr-NIRt data fusion method based on NIRS instrument alone provide a lower cost and a higher identification efficiency than other data fusion techniques without combining with other instruments.

In previous studies, NIRr has been mainly reported for the non-destructive detection of internal mildewing of the seeds [10,11], since it is able to evaluate the main mildew characteristics, namely, mold contamination and the change in chemical composition, from around the surface layer of seed kernels. Despite not targeting the surface of the kernels, NIRt allows for the analysis of optical path depth information accumulation, thereby providing information about the internal structure of the seed kernels [34]. Compared with NIRr, NIRt can also reduce the interference from the outer shell and stray light [35]. On the other hand, our research reveals that the spectral quality of NIRr is better than that of NIRt over the long wavelength range, and vice versa in the short wavelength range. From mid-infrared analysis results, it can be seen that the characteristic absorption peaks of complementary regions are closely related to the main mildew marker metabolites. 

Compared with single spectrum, NIRr-NIRt spectrum fusion technology could effectively realize the classification accuracy of different mildew degrees, especially the slightly moldy degree, based on the synergistic advantages of complementary or enhanced signals of the two spectra.

In addition, the 1D-CNN model produced better classification results than other traditional learning methods. We thus conclude that a deep learning method with stronger feature learning and extraction capabilities is more suitable for analyzing the complicated fusion near-infrared spectral data than shallow learning methods. Thus, the combination of NIRr-NIRt fusion spectra and the 1D-CNN obtained the best performance.

## 5. Conclusions

In this study, the potential of the NIRr-NIRt fusion spectra coupled with a 1D-CNN was evaluated for its ability to non-destructively classify the internal mildew grades of shelled sunflower seeds. To this end, sunflower seeds were divided into three grades of internal mildew (normal, slightly moldy, and seriously moldy) using a reasonable evaluation standard. Precisely because of the differences in microbial count and metabolites among the different mildew grades, NIR was able to capture the information needed to build a reliable discriminant model. Subsequently, the NIRr-NIRt fusion spectra was confirmed to be capable of providing a better discrimination result than single spectra. Following this, the spectral characteristics of sunflower seeds with different mildew grades were effectively analyzed using 1D-CNN with FD pretreatment further improving this effect. The fusion spectral model based on 1D-CNN yielded a prediction accuracy of 97.60% and an F1 score of 97.63% for the training set, and a prediction accuracy of 94.04% and an F1 score of 93.93% for the test set, both of which were superior to those of the single spectra. The results also indicate that the CNN model with strong feature extraction and learning ability yielded better recognition performance than SVM, and KNN models and slightly better than ANN model. These results demonstrate that this method represents a promising alternative for the non-destructive classification of internally moldy sunflower seeds based on NIRS. In future studies, the NIRr and NIRt data fusion method should be implemented at other grades (e.g., medium and high grades). Furthermore, more effective algorithms for the optimal extraction of data could also be developed.

## Figures and Tables

**Table 1:** Characteristics of internally mildewed sunflower kernels with different mildew grades.

No.	Mildew Grade	Visual Grading Standards
1	Normal	Off-white or slightly gray color, uniform, and glossy
2	Slightly moldy	Locally black or brown spots with a mold damage area of less than 50%
3	Seriously moldy	Locally black or brown spots with a mold damage area greater than 50%, obvious shrinkage, and even a loss of the surface characteristics of sunflower seeds

Note: the mildew grade was determined by the area of mold damage as a percentage of the kernel.

**Table 2:** The basic architecture and main parameter settings of the 1D-CNN model for the fusion and single spectral datasets.

Layers	Model Parameters
Input layer (I1)	NIRS data
Conv 1D (C2)	Kernel size = 3, strides = 1, filters = 64, the ReLU function
MaxPooling (S3)	Pooling size = 3,
Conv 1D (C4)	Kernel size = 3, strides = 1, filters = 64, the ReLU function
MaxPooling (S5)	Pooling size = 3
Flatten (F6)	Flatten the feature vector of S5 layer into 1 vector
Dense (F7)	64 Output neurons fully connected to all neurons in layer *F*6, the ReLU function
Dense (F8)	3 Output neurons fully connected to all neurons in layer *F*7, the ReLU function
Output layer	The softmax function

**Table 3:** Discriminant effects of NIRr-NIRt fusion spectral and single spectral 1D-CNN model under different pretreatment methods.

Spectral Data	Pretreatment Method	Train-PA (%)	Train-F1 Score (%)	Test-PA (%)	Test-F1 Score (%)
NIRr	NP	86.82 ± 2.01	85.59 ± 1.84	79.35 ± 1.57	78.68 ± 1.61
	FD	93.72 ± 0.64	93.66 ± 0.59	90.71 ± 0.87	90.44 ± 0.96
	SNV	91.00 ± 0.6	90.58 ± 0.72	83.89 ± 0.96	82.87 ± 0.98
NIRt	NP	78.60 ± 3.49	78.97 ± 0.83	71.91 ± 3.03	69.87 ± 0.33
	FD	89.28 ± 0.47	89.16 ± 0.48	86.58 ± 1.27	86.92 ± 1.39
	SNV	90.78 ± 1.17	90.69 ± 1.18	82.22 ± 1.09	82.04 ± 1.03
Fusion NIRr-NIRt	NP	94.54 ± 0.77	94.52 ± 0.76	80.50 ± 1.25	80.00 ± 1.29
	**FD**	**97.60 ± 0.60**	**97.63 ± 0.69**	**94.04 ± 0.65**	**93.93 ± 0.60**
	SNV	97.48 ± 0.44	97.46 ± 0.45	84.98 ± 0.33	84.72 ± 0.45

Note: The experimental group with the best results is indicated in bold. Abbreviations: 1D-CNN, one-dimension convolutional neural network; NIRr, near-infrared diffuse reflectance; NIRt, near-infrared diffuse transmission; FD, first derivative; SNV, standard normal variable transformation; NP, no preprocessing.

**Table 4:** Prediction results of the 1D-CNN and the other methods.

Algorithm	Train PA (%)	Train F1 Score (%)	Test PA (%)	Test F1 Score (%)
CNN	97.60 ± 0.60	97.63 ± 0.69	94.04 ± 0.65	93.93 ± 0.60
ANN	96.60 ± 0.21	97.52 ± 0.16	92.03 ± 0.35	91.95 ± 0.31
SVM	92.08	92.63	88.07	87.75
KNN	91.4	91.08	83.49	83.67

Abbreviations: PA, prediction accuracy; CNN, convolutional neural network; ANN, artificial neural network; SVM, support vector machine; KNN, K-nearest neighbor.

## Data Availability

The data during the current study are available from the corresponding author on reasonable request.
